# Perspective: the role of science in society

**DOI:** 10.1093/af/vfae035

**Published:** 2025-04-05

**Authors:** Keith E Belk, John A Scanga, Mahesh N Nair, Peipei Zhang, Gina Geornaras, Robert J Delmore

**Affiliations:** Center for Meat Safety & Quality, Colorado State University, Fort Collins, CO, USA; Center for Meat Safety & Quality, Colorado State University, Fort Collins, CO, USA; Center for Meat Safety & Quality, Colorado State University, Fort Collins, CO, USA; Center for Meat Safety & Quality, Colorado State University, Fort Collins, CO, USA; Center for Meat Safety & Quality, Colorado State University, Fort Collins, CO, USA; Center for Meat Safety & Quality, Colorado State University, Fort Collins, CO, USA

**Keywords:** research, science, scientific method, scientist, society

**Implications**

• The term ‘science’ is used for many purposes, sometimes in error. Generally, ‘science’ is “use of evidence to construct testable explanations and predictions of natural phenomena, as well as the knowledge generated.” New scientific knowledge is subjected to peer review by discipline, a process leading to quality assurance. Science approaches becoming fact as consensus is reached, almost always via redundant study.

• Although science benefits mankind, it is often maligned. Those not trained in science lack credibility in their criticism, but they wish to exert control where scientific results depart from their convictions. Some prioritize convictions as more important than evidence, so they attempt to undermine science; they even attack the reputation of scientists. Neither is acceptable.

• To advance, society must better understand science. Scientists themselves must be truthful and differentiate personal convictions from objective results of investigation. A scientist’s primary role is not to modify value systems in society—only to inform them.

• Only when these things occur can misinformation in society be addressed.

## Introduction

‘Science’ has profoundly improved the quality of life for mankind. Although a noble endeavor, it seems that the term is often used without a true understanding of its meaning, which leaves opportunities for abuse. Special interest groups frequently denigrate the term. In fact, due in part to societal ignorance, science can be compromised by misinformation and agendas. Such is true for applied science addressing meat and livestock issues of importance. Misinformation via social media and other avenues can serve to indoctrinate and shift public understanding. News media can become complicit in this activity due to a lack of academic understanding. And because media historically were a primary conduit for communicating science to the public, the media also can sometimes unintentionally advocate for misinformation. Likewise, ideological activism and indoctrination have occurred disguised as ‘science’; this too is inappropriate. The goal of this paper is to explore science (with emphasis on animal agriculture) and society.

## What is Science?

If one searches for definitions of the term ‘science’, a plethora of options are provided. Even across the world’s most popular dictionaries, no definitions seem to be the same. However, according to the National Academies Institute of Medicine (Institute of Medicine, 2008), ‘science’ is defined as:

*“The use of evidence to construct testable explanations and predictions of natural phenomena, as well as the knowledge generated through this process.”*

Further, and as alluded to in this definition, the archaic meaning of ‘science’ was “a comprehensive and articulated knowledge” (Leydi, 2022) or the ‘total body of knowledge’. Over time, that meaning has morphed to include systematic collections of knowledge and/or methods (Leydi, 2022). In short, to say that something is ‘science-based’ implies that the information was derived using testable explanations and reflects the current body of existing knowledge.

## What is Research?

The term ‘research’ can be used as a noun or a verb with different meanings depending on context. As a verb, it refers to a systematic process of establishing new knowledge. Thus, ‘research’ generically describes how knowledge is created or expanded.

## What is the Scientific Method?

The ‘scientific method’ is the operating procedure by which scientific research occurs. However, the ‘scientific method’ is not standardized; it generally entails an approach to investigating a question regarding the unknown that is objective, logical, and agreeable to those within a scientific discipline.

Then how is the correct application of the ‘scientific method’ judged? Unlike with other forms of business, law, or art, information derived via the scientific method has entailed centuries of advancement leading to a process of transparency and peer review (i.e., scientific rigor); peers among a discipline pass judgment on the suitability and accuracy of evidence-based conclusions.

As described in the **Stanford Encyclopedia of Philosophy** (2021), “Among activities often identified as characteristic of science are systematic observation and experimentation, inductive and deductive reasoning, and the formation and testing of hypotheses and theories.” Hence, in general, the ‘scientific method’ includes seven activities: (1) Define a question, (2) Assess literature to determine the current state of knowledge, (3) Establish null and alternative hypotheses, (4) Design and conduct an experiment, (5) Use deductive reasoning, statistics, and other tools to analyze data, (6) Formulate conclusions and implications, and (7) Prepare a report, subject it to peer-review, and publish. Over time, repetition of this process allows the formation of scientific consensus—which implies that the trained scientific community considers the consensus knowledge as factual.

Science followed by peer review is not a perfect system, but it is the most objective so far. Alas, the National Academies of Sciences, Engineering, and Medicine (2019) declared that “Uncertainty is inherent in all scientific knowledge, and many types of uncertainty can affect the reliability of a scientific result... Decision makers looking to use study results need to be able to understand the uncertainties associated with those results” and “When results are computationally reproduced or replicated, confidence in the robustness of the knowledge derived from that particular study is increased.”

## What is a Scientist?

For purposes here, a ‘scientist’ is defined as a professional who is trained and knowledgeable in the application of the scientific method. Evidence that a person is such a professional can be provided by having a PhD or analogous degree. All those with PhDs are not necessarily scientists (and vice versa), but the Ph.D. validates exposure to, and mentorship in, the application of the ‘scientific method’ in a given discipline. Someone untrained in the ‘scientific method’ is unfamiliar with the process of systematic study of a question leading to the formulation of conclusions and, therefore, has reduced credibility. In contrast, would you hire someone to defend you in court who was not trained in the law? Would you request a health diagnosis from someone not trained in medicine? Hence, experts who review and criticize science should be knowledgeable of the accepted scientific method in the discipline under consideration.

## Science, Policy, and the Public

Knowledge derived by science informs societal behaviors and policies. Furthermore, technologies discovered via science can be circular in that they can improve the next round of investigation and can result in better lifestyles for mankind. But how science is incorporated into life by society is sometimes contentious, leading to polarization based on ideology. Although discourse on the use of science is necessary and good, forces of misinformation, disinformation, and outright lies often manifest themselves seeking to promote a particular agenda or preference. It is at the interface of how science is to be used that an important question arises: should the scientist be a disinterested investigator and communicator of conclusions related to the previously unknown, or should the scientist align their pursuit of knowledge and outreach with prevailing agendas and ideologies?

To answer this question, the National Academies Institute of Medicine (2009) said that: “Researchers have a professional obligation to perform research and present the results of that research as objectively and as accurately as possible. When they become advocates on an issue, they may be perceived by their colleagues and by members of the public as biased. But researchers also have the right to express their convictions and work for social change, and these activities need not undercut a rigorous commitment to objectivity in research.” However, the reverse of objectivity can also occur (Ederer, 2024), and sometimes pseudo-science has been compromised by activism.

Thorp (2024) wrote in a recent *Science* editorial piece that: “a document called the Kalven Report that was produced at the University of Chicago in 1967 famously declared that ‘the university is the home and sponsor of critics; it is not itself the critic.’ Thus, in matters of political controversy, the university best serves its faculty and students by remaining neutral so that those with disciplinary expertise can opine freely.” Although there was more to Thorp’s (2024) editorial than indicated here, he went on to cite Dartmouth College President Sian Beilock as reflecting: “I think a scientist’s job is to do the best work they can and publish the findings that they believe will be most impactful to humanity... regardless of whether their conclusions align with one particular idea.”

A preface to a National Academies of Sciences, Engineering, and Medicine (2019) report on *Reproducibility and Replicability in Science* stated that: “When a scientific study becomes the basis of policy or has a direct or indirect impact on human well-being, scientific reliability becomes more than an academic question.”

Recently, social scientists characterized the nature of damage in society resulting from misinformation rather than science, likening severe outcomes to those resulting from the tobacco industry when human health concerns developed from smoking (van der Linden and Krychenko, 2024). The authors stated that “It is therefore important to have realistic expectations for standards of evidence” when science is used in public policy, and that future research should try to understand the impact of “super sharers” of misinformation because they have the potential to cause “outsized harm.” It seems that ‘adequacy of science’ is sometimes confused with ‘effectiveness of scientific communication.’

According to West and Bergstrom (2021), a great many “analyses of misinformation focus on popular and social media, but the scientific enterprise faces a parallel set of problems—from hype and hyperbole to publication bias and citation misdirection, predatory publishing, and filter bubbles”. To reduce exposure to misinformation, they stated that: “Public engagement and understanding of science should be a priority for all scientists... it involves nurturing innate curiosity and teaching people to understand how science works, how to consider evidence when making conclusions, and how popular media distorts these conclusions.” Although the public does not require a substantial understanding of the ‘scientific method’, improved familiarity would help to overcome the consequences of misinformation.

Scientists must exert robust peer-review scrutiny of studies to be published if credibility is to exist. Information derived from research that is disseminated in an environment of mistrust without explanation of complex systems will generate “outrage” (Hooker et al., 2017). As described by the International Atomic Energy Agency (2012) following the meltdown of the Japanese Fukushima nuclear reactor, “Any action or communication that damages trust, such as delayed, withheld, or misleading information, will raise public apprehension and actively contribute to increased risk to public health and well-being.”

Regarding scientific incompetence, Ederer (2024) used three prominent case examples to describe how agenda-driven scientists can gravely distort the objectivity of science and do considerable harm to society as their desired agendas turn out to be false. The three examples included the WHO-IARC findings on red meat being associated with cancer, the Global Burden of Disease studies emphasizing unfounded dangers of red meat while dismissing its benefits, and the proceedings at the Global Food Systems Summit in 2021, where false science was deployed to promote an anti-livestock global policy agenda. In all examples, authors did not follow through with reason (because it apparently violated their agenda) as they published conclusions that were contrary to their own discussion of data. Or, they had clear conflicts of interest regarding the credibility of their motives and findings (Ederer, 2024). It was noteworthy that the first-of-its-kind Dublin Declaration (2022), now signed and vetted globally by 1,210 scientists, stated in the very first sentence that: “Livestock systems must progress on the basis of the highest scientific standards. They are too precious to society to become the victim of simplification, reductionism, or zealotry.” However, it seems that agenda-driven science does not play by the same rules; society should question it as new values are discovered.

Likewise, science should not be undermined by, nor should credence be given to, ideological attacks from unqualified activists, or by attacks directly on the scientists themselves (e.g., Viveca and Jacquet, 2024). If civilization is to advance, scientific consensus must be considered in policy independently to inform the development of societal values. Albert Einstein was credited with saying (Internet Sacred Text Archive, 1941) that “Science can only ascertain what is, but not what should be, and outside of its domain value judgments of all kinds remain necessary.” Karl Popper, a well-recognized epistemologist, remarked: “There is no criterion for truth at our disposal... but we possess criteria which, if we are lucky, may allow us to recognize error and falsity.” (Popper, 1960).

## Conclusions

So, to maintain and increase the benefits of science to society, it is necessary for scientists to 1) help society understand the science, 2) tell the truth as it is currently known, 3) do not presume definitive convictions and indoctrinate based on personal values, 4) serve in a primary role of sharing knowledge, and 5) seek new knowledge. In return, non-scientists need to resist short-term agendas and a temptation to interfere with the scientific processes and its institutions, as well as the evolution of scientific consensus, for the sake of maintaining the long-term integrity of this pillar of progress for humanity.

## About the Authors

Keith Belk serves as Professor, Holder of the Monfort Chair, and Director of the Center for Meat Safety & Quality, at Colorado State University (CSU). Previously, he served as Head of the Department of Animal Sciences at CSU. He earned B.S. and M.S. degrees from CSU and a Ph.D. from Texas A&M University. He has been employed as a buyer by Safeway, Inc., and by the USDA Agricultural Marketing Service in Washington, DC, as an International Marketing Specialist. At CSU since 1995, he has authored or coauthored >260 refereed scientific journal articles and generated >$22M in research funding. He served as President of AMSA from 2009 to 2010, and currently serves on the International Committee for the National Western Stock Show, the Board of Directors for the International Stockmen’s Educational Foundation, on the Academic Advisory Committee for the North American Meat Institute Protein PACT, and on the Global Technical Advisory Committee for JBS. **Corresponding author**: keith.belk@colostate.edu

Dr. John Scanga received his PhD in Animal Sciences from Colorado State University in Fort Collins, CO. He and his wife Chauna and their sons Sam and Ian reside in Ault, CO, and operate a diversified farming operation. His experience includes Associate Professor in the Center for Meat Safety and Quality at Colorado State University, Vice President of Technical Services for IEH Laboratories, Technical Consultant at Elanco Animal Health, and Vice President of Quality Assurance and Procurement at Meyer Natural Foods. John currently serves as a Visiting Professor in the Department of Animal Sciences at Colorado State University and as Chief Scientific Officer for Eurofins Laboratories North American Meat Division. He has served his community and the industry as President of his local school board, served as an AMSA director, and is currently Chair of the Colorado Beef Council.

Mahesh Nair is an Associate Professor of Meat Science in the Department of Animal Sciences at Colorado State University. He received his BS in Veterinary Medicine from Kerala Agricultural University, India (2009), and MS (2012) and PhD (2017) from the Department of Animal and Food Sciences at the University of Kentucky. Dr. Nair’s research focuses on the fundamental and applied aspects of fresh meat quality, with an emphasis on meat color and shelf-life. His other research interests include postmortem muscle biochemistry, the role of mitochondria in meat quality, as well as the interactions between myoglobin redox stability and lipid oxidation. He has authored or coauthored more than 70 peer-reviewed journal articles and 5 book chapters, in addition to several abstracts at scientific conferences. At CSU since 2017, he has generated more than $4 million in research funding and has served on 30 graduate committees either as a chair or committee member.

Peipei Zhang is an Assistant Professor of food microbiology in the Department of Animal Sciences at Colorado State University. She obtained her PhD in Agricultural Sciences at the University of Tasmania in Australia and did her postdoctoral research at Agricultural and Agri-Food Canada. Peipei Zhang has been doing research in the area of meat microbiology and safety. Her research interests include green/sustainable technologies to improve meat safety and extend meat shelf life and the microbial ecology in meat production environments. She is also an active member of professional organizations including the International Association for Food Protection, and the American Meat Science Association. So far, she has 25 publications in peer-reviewed journals such as the International Journal of Food Microbiology, Applied and Environmental Microbiology, and Food Research International, receiving 404 citations with a h-index of 11.

Dr. Gina Geornaras is a Professor in the Department of Animal Sciences at Colorado State University. She received her BS (Microbiology and Genetics), BS Honors (Microbiology), and PhD (Food Microbiology) from the School of Molecular and Cell Biology at the University of the Witwatersrand, South Africa. Since joining Colorado State University in 2002, she has collaborated with faculty members, postdoctoral fellows, graduate students, and visiting scientists of the Center for Meat Safety & Quality on research projects focused on microbial meat quality and the ecology, detection, and control of bacterial pathogens in foods. Additionally, Dr. Geornaras coordinates and supervises activities in the department’s food safety/microbiology and food chemistry laboratories.

Dr. Robert Delmore is a professor in the Department of Animal Sciences. He teaches courses in Meat Science, Meat Processing, and Food Safety. He is a member of the Center for Meat Safety & Quality conducting research to address national and global issues. He recently oversaw the design and construction of the JBS Global Food Innovation Center in Honor of Gary and Kay Smith. He directs the production of Beef Sticks for Backpacks, a nonprofit providing high-quality beef sticks to food-insecure children in Colorado. In June 2022 Bob was designated as a Fellow in the American Meat Science Association. Bob and his wife Dr. Lynn Delmore, also a well-regarded meat scientist, live in Wellington with their two daughters Ainsley and Tessa.
